# Supplementary material for: Extracellular fluid viscosity enhances cell migration and cancer dissemination
Source: Nature. 2022 Nov 2;611(7935):365–73. doi: 10.1038/s41586-022-05394-6 (PMC9646524; doi:10.1038/s41586-022-05394-6)
Supplement: Supplementary file 1 — Supplementary Fig. 1: uncropped images of western blots presented in the manuscript. The dashed red rectangles indicate the areas that were cropped and displayed in the figures. [file 41586_2022_5394_MOESM1_ESM.pdf]

nature portfolio

https://doi.org/10.1038/s41586-022-05394-6

Supplementary information

Extracellular fluid viscosity enhances cell migration and cancer dissemination

In the format provided by the authors and unedited

Supplementary Figure 1

Uncropped blots for shARP3 and shARPC4 (*used in Extended Data Fig. 4b*)

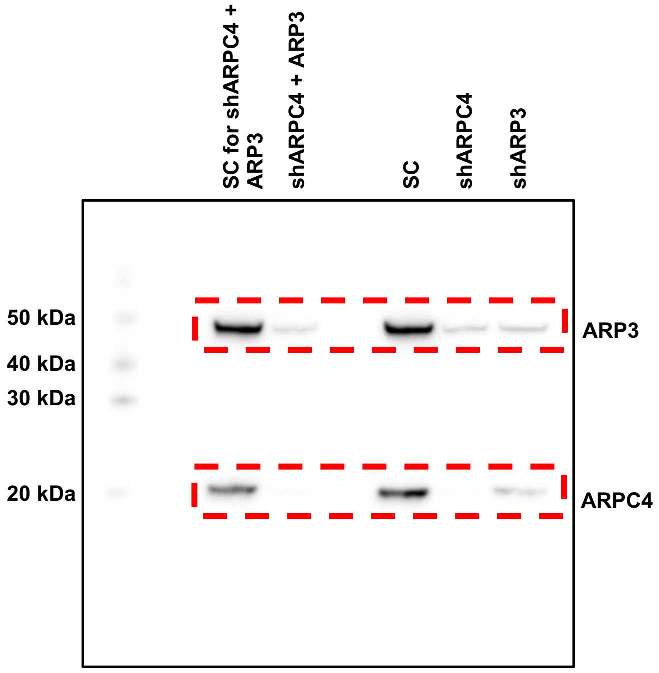

3 sec

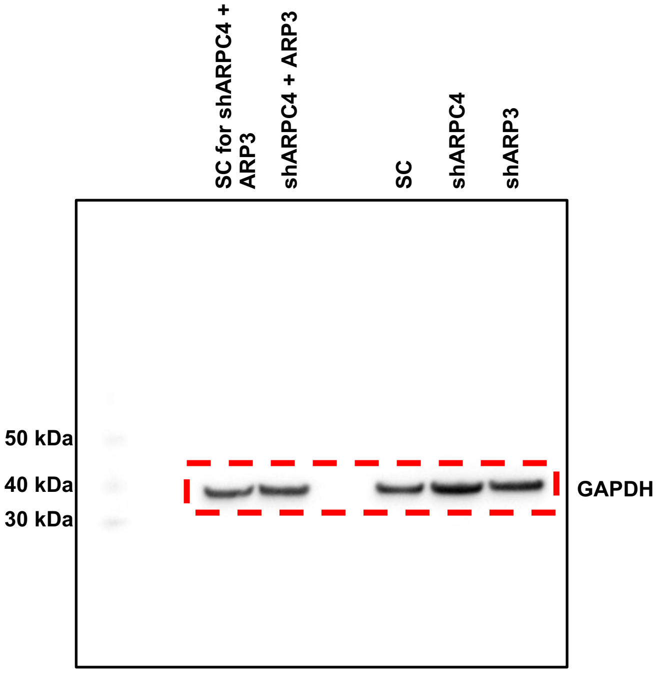

2 sec
(after stripping and reblotting)

All the lanes imaged were run in one single gel. GAPDH was imaged after stripping and reblotting the membrane used for blotting ARP3.

Uncropped blots for shNHE1 (*used in Extended Data Fig. 5a*)

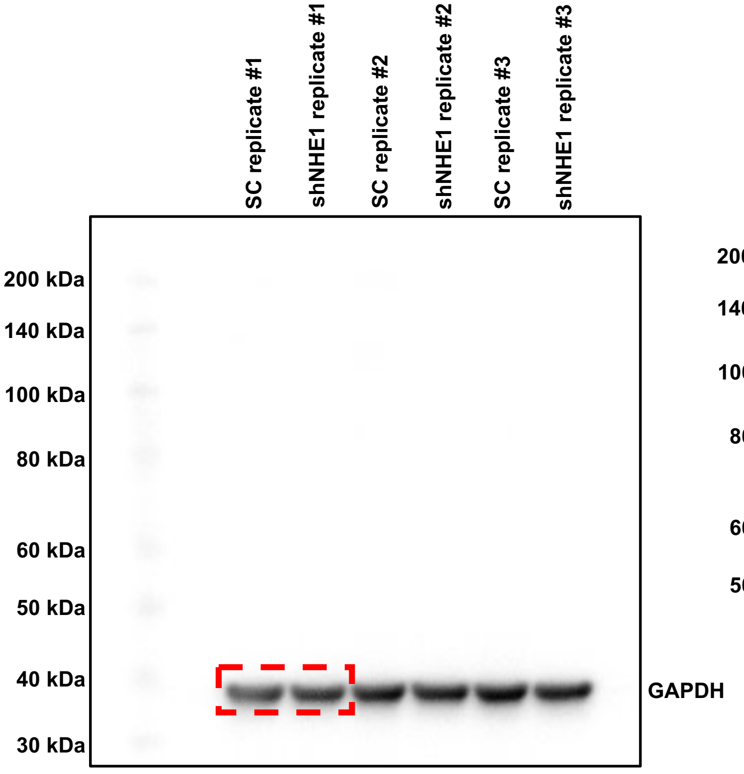

1 sec

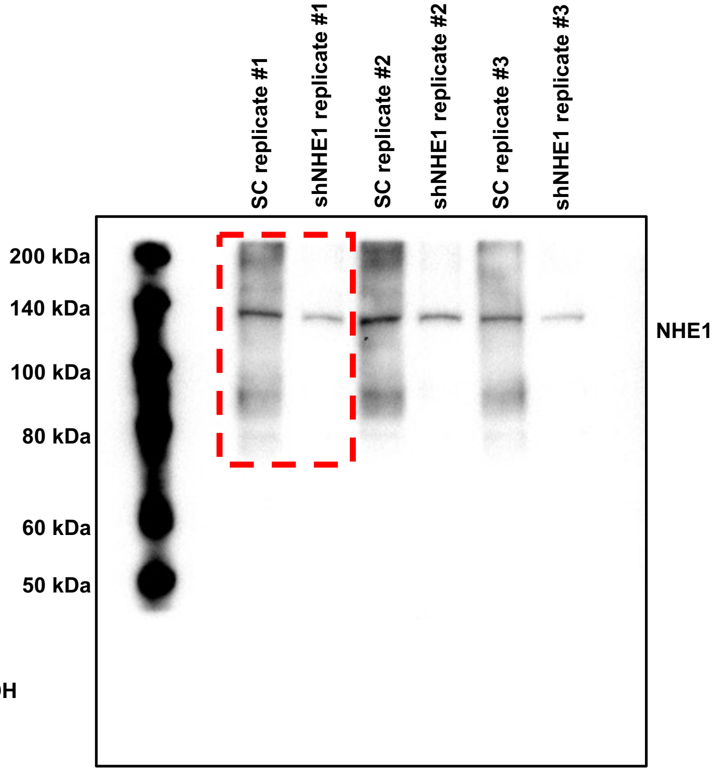

153 sec

All the lanes imaged were run in one single gel.

Uncropped blots for NHE1 shRNA1 & shRNA2 (*used in Extended Data Fig. 5g*)

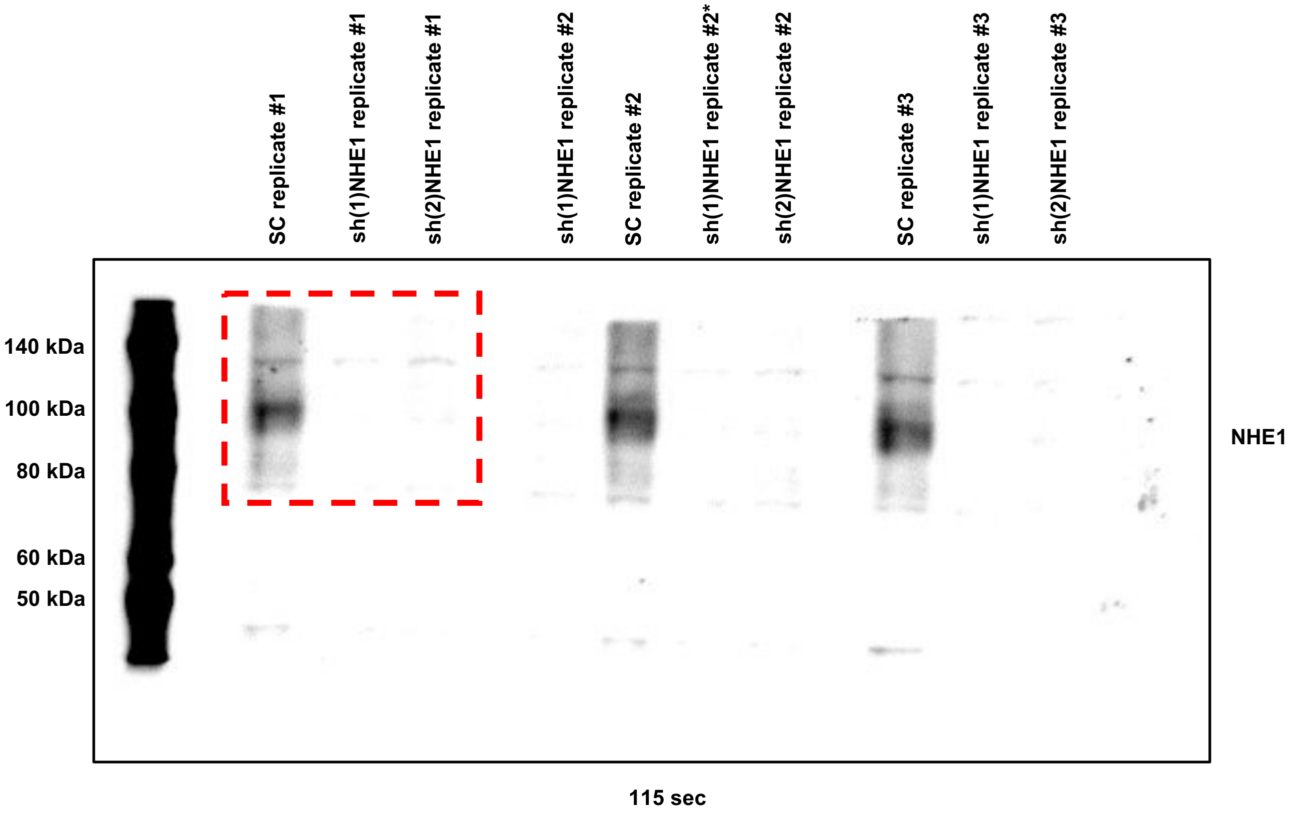

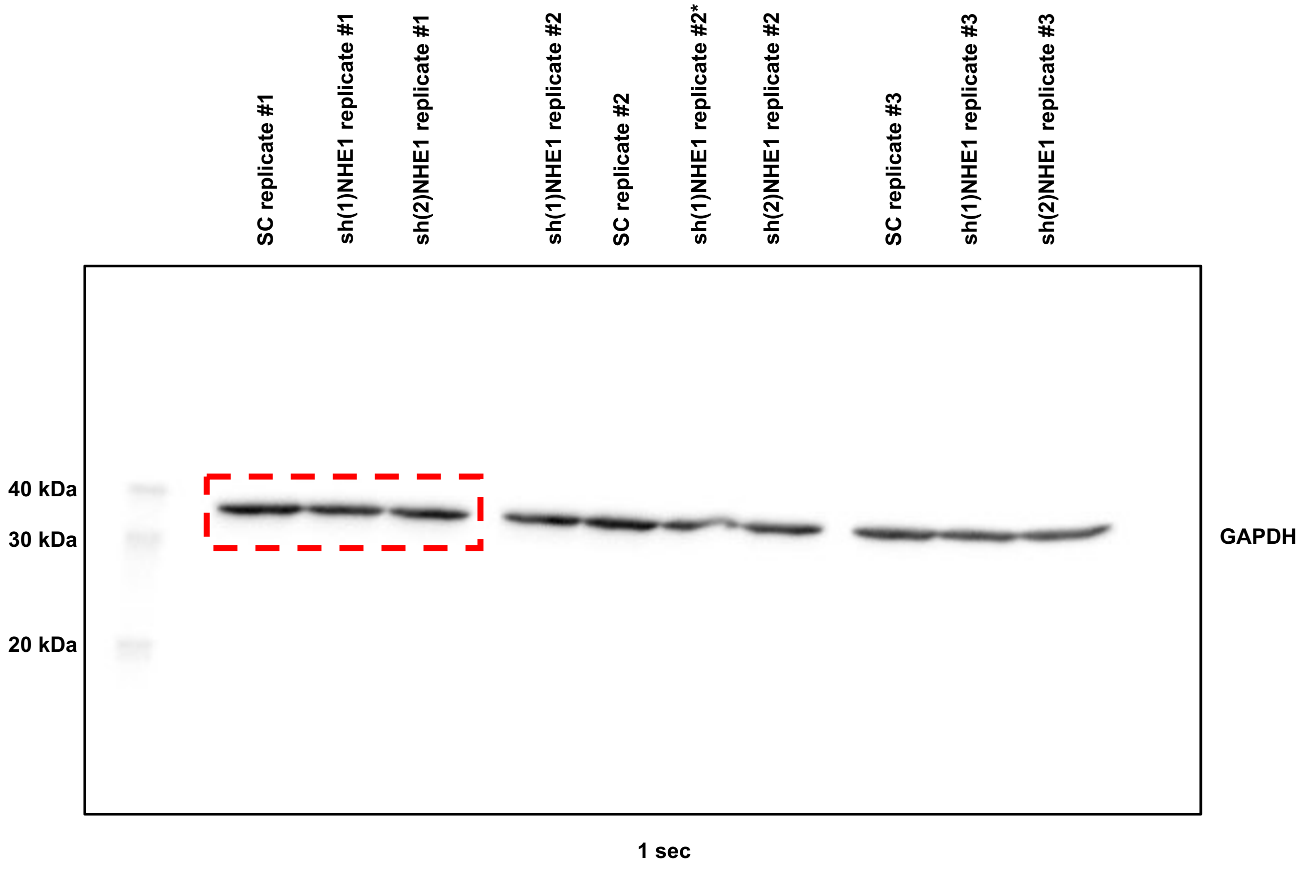

All the lanes imaged were run in one single gel.

Uncropped blots for shTRPV4 (*used in Extended Data Fig. 6a*)

a

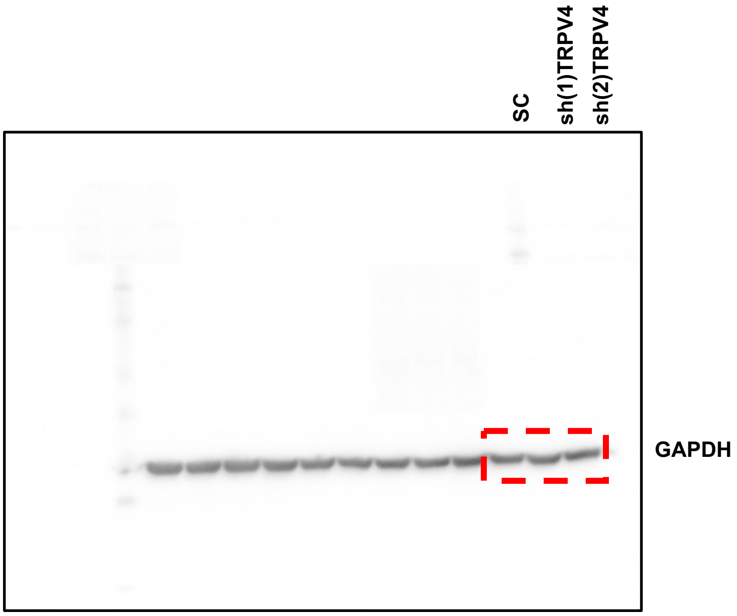

1 sec

b

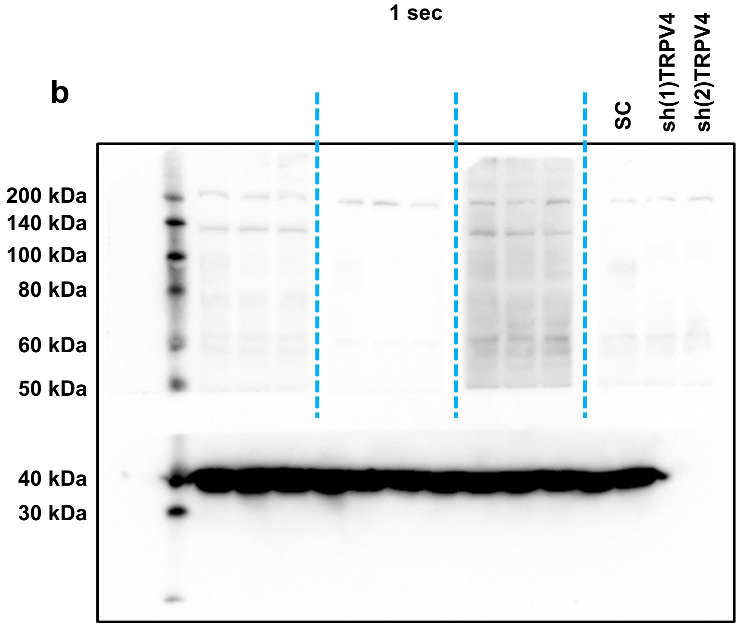

15 sec

c

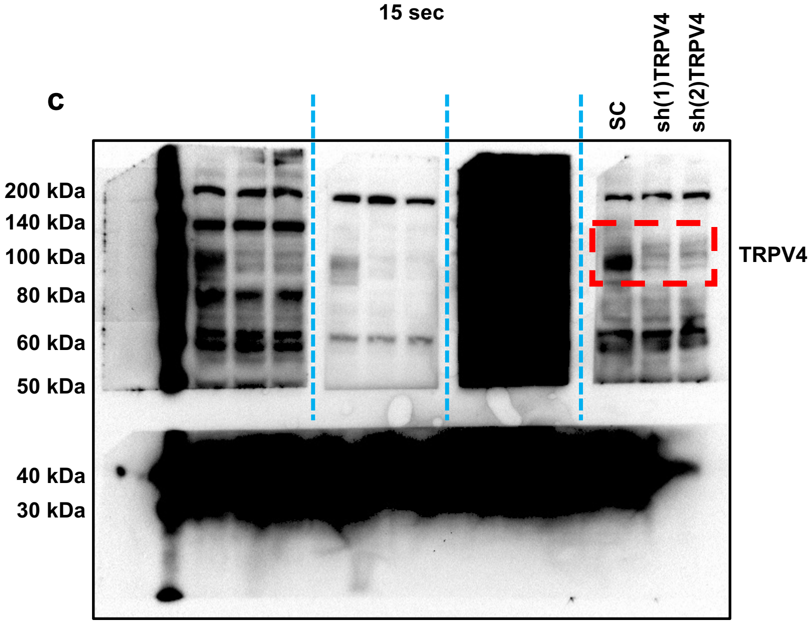

300 sec

All the lanes imaged were run in one single gel. The top part of the membrane, after protein transfer from the gel, was cut vertically (along the blue dotted lines) and the membranes were imaged at 15 sec and 300 sec exposures to visualize the molecular weight ladder and TRPV4, respectively. In (b) and (c) the membrane pieces were spaced apart during imaging to prevent interferences from adjacent pieces.

Uncropped blots for shITGB1 (*used in Extended Data Fig. 9a*)

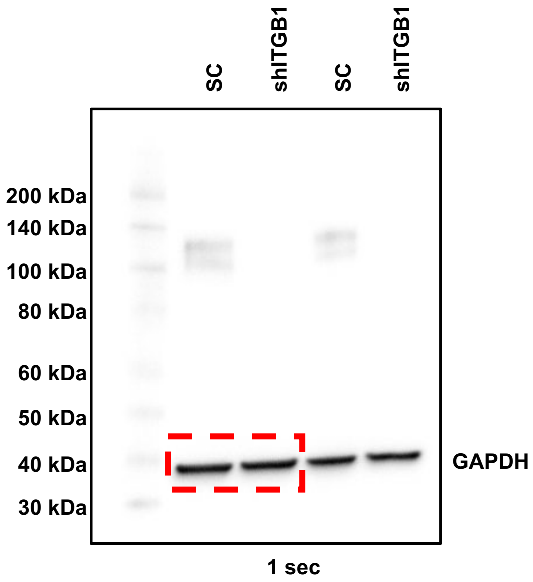

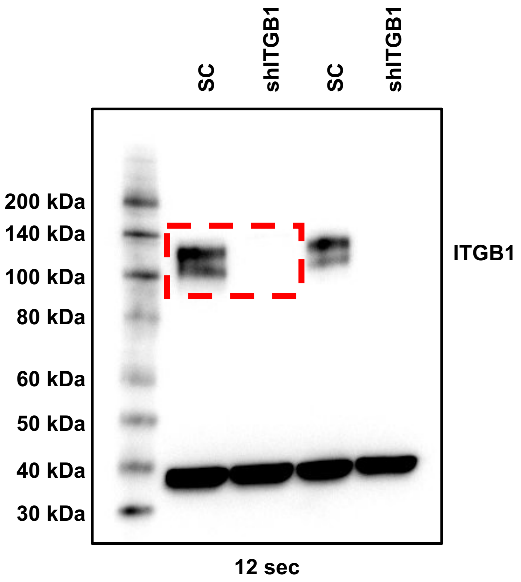

All the lanes imaged were run in one single gel.
