# Supplementary material for: Extracellular fluid viscosity enhances cell migration and cancer dissemination
Source: Nature. 2022 Nov 2;611(7935):365–73. doi: 10.1038/s41586-022-05394-6 (PMC9646524; doi:10.1038/s41586-022-05394-6)
Supplement: Supplementary file 2 — Reporting Summary [file 41586_2022_5394_MOESM2_ESM.pdf]

nature portfolio

Corresponding author(s): Konstantinos Konstantopoulos (konstant@jhu.edu)

Last updated by author(s): Jul 28, 2022

Reporting Summary

Nature Portfolio wishes to improve the reproducibility of the work that we publish. This form provides structure for consistency and transparency in reporting. For further information on Nature Portfolio policies, see our Editorial Policies and the Editorial Policy Checklist.

Statistics

For all statistical analyses, confirm that the following items are present in the figure legend, table legend, main text, or Methods section.

n/a	Confirmed	
☐	☒	The exact sample size (*n*) for each experimental group/condition, given as a discrete number and unit of measurement
☐	☒	A statement on whether measurements were taken from distinct samples or whether the same sample was measured repeatedly
☐	☒	The statistical test(s) used AND whether they are one- or two-sided<br>*Only common tests should be described solely by name; describe more complex techniques in the Methods section.*
☐	☒	A description of all covariates tested
☐	☒	A description of any assumptions or corrections, such as tests of normality and adjustment for multiple comparisons
☐	☒	A full description of the statistical parameters including central tendency (e.g. means) or other basic estimates (e.g. regression coefficient) AND variation (e.g. standard deviation) or associated estimates of uncertainty (e.g. confidence intervals)
☐	☒	For null hypothesis testing, the test statistic (e.g. *F*, *t*, *r*) with confidence intervals, effect sizes, degrees of freedom and *P* value noted<br>*Give P values as exact values whenever suitable.*
☒	☐	For Bayesian analysis, information on the choice of priors and Markov chain Monte Carlo settings
☒	☐	For hierarchical and complex designs, identification of the appropriate level for tests and full reporting of outcomes
☒	☐	Estimates of effect sizes (e.g. Cohen's *d*, Pearson's *r*), indicating how they were calculated

Our web collection on statistics for biologists contains articles on many of the points above.

Software and code

Policy information about availability of computer code

Data collection	Data were collected using standard features of NIS-Elements (Version 5.02.01 or Version 3.22.00), ImageJ (1.53f51), Axon p-Clamp (Version 10), Hamamatsu HCImage (v1.0, 2018), ZEN 2.3 SP1 FP3 (black), SymPhoTime 64 (Version 2.4) or ZEN 2.3 (blue edition).
Data analysis	Data were organized primarily using Microsoft Excel (Version 16.57) and Graphpad Prism (Version 7,8 or 9). Select analysis were performed in MATLAB (Version R2021b, 9.11.0.1809720), SigmaPlot (Version 10), NIS-Elements (Version 5.02.01), Picasso (Version 0.3.8), SymPhoTime 64 (Version 2.4), Imaris (Version 9.7.0), HISAT2 (Version 2.2.1), HTSEQ (Version 0.13.5) or DEseq2 (Version 1.30.1, using R 4.0 version), as described in Methods. The human GRCh38.p13 genome (hg38) was obtained from Ensembl (release 104). The code used for data analysis and stochastic actin network simulation is available on GitHub in a link provided by KK: https://github.com/KKLabJHU. The code for the two-phase model is available on GitHub in a link provided by SXS: https://github.com/sxslabjhu/

For manuscripts utilizing custom algorithms or software that are central to the research but not yet described in published literature, software must be made available to editors and reviewers. We strongly encourage code deposition in a community repository (e.g. GitHub). See the Nature Portfolio guidelines for submitting code & software for further information.

Data

Policy information about availability of data

All manuscripts must include a data availability statement. This statement should provide the following information, where applicable:

- Accession codes, unique identifiers, or web links for publicly available datasets
- A description of any restrictions on data availability
- For clinical datasets or third party data, please ensure that the statement adheres to our policy

The main data supporting the results of this study are available within the paper and its Extended Data Figure file. All source data are provided with this paper.

Supplementary Information is also included for this paper. RNA-sequencing data are available at the National Center for Biotechnology Information Gene Expression Omnibus, under accession number GSE203651.

Field-specific reporting

Please select the one below that is the best fit for your research. If you are not sure, read the appropriate sections before making your selection.

☒ Life sciences ☐ Behavioural & social sciences ☐ Ecological, evolutionary & environmental sciences

For a reference copy of the document with all sections, see nature.com/documents/nr-reporting-summary-flat.pdf

Life sciences study design

All studies must disclose on these points even when the disclosure is negative.

Sample size	We indicated in supplementary information the exact sample size, number of replicates and p value for each experiment. No predetermination of sample size was done. Sample size was chosen based on the throughput of the technique used. Sample sizes were sufficient to show the same trends between the replicates performed for each experiment, and by statistical testing. For animal experiments, no statistical methods were used to calculate sample size and group size, and the sample size was determined based on experience of similar assays performed earlier. In each experiment multiple cells or animals were examined in parallel leading to sample sizes primarily of the order of tens to hundreds.
Data exclusions	Data were excluded for in vitro migration experiments based on pre-established criteria also mentioned in the Methods section: dividing or apoptotic cells were excluded from analysis. For mice experiments, animals in which the entire volume of cancer cell suspension was not successfully injected into the mouse tail vein were immediately excluded from the study. For zebrafish experiments, fish lacking human cells, as identified from microscopic screening ~1-4h post injection, were excluded from imaging. During analysis of cell trajectories in intersegmental vessels, only cells with a diameter of at least 10 µm were tracked to avoid cell fragments. Speeds and persistences were calculated only for cells remaining in the intersegmental vessels for at least 4 frames.
Replication	The exact number of replicates for each experiment has been indicated in the figure legends and supplementary information. Most experiments were repeated 3 or more times, with similar results observed each time. Select control experiments were repeated 2 times with consistent data across all replicates.
Randomization	For zebrafish experiments, fish from a given clutch were randomly divided into experimental groups prior to injection. For CAM extravasation assays, embryos were randomly divided into experimental groups prior to injection. Mice were randomized based on weight to maintain similar average weight across experimental groups. For all other experiments, cells were randomly distributed into experimental groups before imaging and analysis.
Blinding	RNA sequencing and data analysis and mice injections were performed in a blinded manner, without prior knowledge of cell pre-treatment conditions or identity of shRNA-mediated modifications. For remaining experiments researchers were not blinded as data collection and analysis were performed by the same individual assigning the groups. Wherever possible findings (e.g., cell migration speeds, cell volume) were analyzed in an unbiased manner by the use of automated Fiji and custom analysis codes in Matlab.

Reporting for specific materials, systems and methods

We require information from authors about some types of materials, experimental systems and methods used in many studies. Here, indicate whether each material, system or method listed is relevant to your study. If you are not sure if a list item applies to your research, read the appropriate section before selecting a response.

Materials & experimental systems

n/a	Involved in the study	
☐	☒	Antibodies
☐	☒	Eukaryotic cell lines
☒	☐	Palaeontology and archaeology
☐	☒	Animals and other organisms
☒	☐	Human research participants
☒	☐	Clinical data
☒	☐	Dual use research of concern

Methods

n/a	Involved in the study	
☒	☐	ChIP-seq
☐	☒	Flow cytometry
☒	☐	MRI-based neuroimaging

Antibodies

Antibodies used	Primary antibodies used for immunostaining were: anti-pMLC (Ser19) antibody (raised in rabbit; Cell Signaling; 3671; Lot# 6; 1:100 dilution); anti-Ki-67 antibody (raised in mouse; clone 8D5; Cell Signaling; 9449; Lot# 4; 1:800 dilution); anti-NHE1 antibody (raised in mouse; clone 54; Santa Cruz Biotechnology; sc-136239; Lot# H2021; 1:50 dilution); anti-Ezrin antibody (raised in rabbit; Cell Signaling; 3145S; Lot# 5; 1:200 dilution); anti-human Vimentin (raised in mouse; clone O91D3; BioLegend; Alexa Fluor 647 conjugated; 677807; Lot# B309436; 1:200 dilution); anti-YAP antibody (raised in mouse; clone 63.7; Santa Cruz Biotechnology;

sc-101199; Lot# G2821; 1:50 dilution). Secondary antibodies used for immunostaining were: Alexa Fluor 488 goat anti-rabbit immunoglobulin G (IgG) H+L, (Invitrogen; A11034; Lot# 2256692; 1:200 dilution), Alexa Fluor 488 goat anti-mouse immunoglobulin G (IgG) H+L, (Invitrogen; A11029; Lot# 2179204; 1:200 dilution) or Alexa Fluor Plus 647 goat anti-mouse immunoglobulin G (IgG) H+L, (Invitrogen; A32728; Lot# WE322197; 1:100 dilution).
Primary antibodies used for western blotting were: anti-TRPV4 antibody (raised in mouse; clone 1B2.6; Millipore Sigma; MABS466; Lot# 3462069; 1:1000 dilution), anti-ARP3 (raised in mouse; FMS338; Abcam; ab49671; Lot# GR234096-12; 1:5000 dilution), anti-ARPC4 (raised in rabbit; Abcam; ab217065; Lot# GR312814-8; 1:2000 dilution), anti-Integrin beta-1 Antibody (raised in rabbit; Cell Signaling; 4706S; Lot#6; 1:1000 dilution) and anti-NHE1 (raised in mouse; clone 54; Santa Cruz Biotechnology; sc-136239; Lot# H2021; 1:200 dilution). GAPDH was used as a loading control (raised in rabbit; 14C10; Cell Signaling; 2118S; Lot# 14; 1:5000 dilution).
Secondary antibodies used for western blotting were: anti-mouse IgG, HRP-linked antibody (Cell Signaling; 7076S; Lot# 36; 1:1000 dilution) and anti-rabbit IgG, HRP-linked antibody (Cell Signaling, 7074S; Lot# 29; 1:1000 dilution).

Validation

Prior to purchasing, antibodies were validated by the manufacturer and available on their website:
https://media.cellsignal.com/coa/3671/6/3671-lot-6-coa.pdf
https://media.cellsignal.com/coa/9449/14/9449-lot-14-coa.pdf
https://datasheets.scbt.com/sc-136239.pdf
https://media.cellsignal.com/coa/3145/5/3145-lot-5-coa.pdf
https://www.biolegend.com/nl-nl/certificate-of-analysis (lot# B309436)
https://datasheets.scbt.com/sc-101199.pdf
https://www.merckmillipore.com/IN/en/product/Anti-Trpv4-Antibody-clone-1B2.6,MM_NF-MABS466#anchor_COA (lot# 3462069)
https://www.abcam.com/arp3-antibody-fms338-ab49671.pdf
https://www.abcam.com/arpc4-antibody-ab217065.pdf
https://media.cellsignal.com/coa/4706/6/4706-lot-6-coa.pdf
https://media.cellsignal.com/coa/2118/14/2118-lot-14-coa.pdf
Additionally, antibodies for western blotting were verified based on the appropriate molecular weight of the protein probed and for immunofluorescence by comparison of their cellular distribution to that provided in the manufacturer's datasheet.

Eukaryotic cell lines

Policy information about cell lines

Cell line source(s)

MDA-MB-231, Human osteosarcoma cells, U87 and HEK cells were purchased from American Type Culture Collection (ATCC). Human dermal fibroblasts (GM05565) were purchased from the Coriell Institute. Human AOSMC was purchased from PromoCell. SUM159 cells were a gift from Denis Wirtz (Johns Hopkins University), brain metastatic MDA-MB-231 (BrM2) cells were from Joan Massagué (Memorial Sloan Kettering Cancer Center). Select cell lines were modified from the parental line, as described in Methods (e.g., development of cell lines with scramble, shRNA or live reporters).

Authentication

Cell lines were originally authenticated by ATCC, Coriell Institute or PromoCell, and were not further authenticated as part of this study.

Mycoplasma contamination

All cell lines were regularly tested by PCR and verified to be free of mycoplasma contamination.

Commonly misidentified lines
(See ICLAC register)

No commonly misidentified cell lines were used.

Animals and other organisms

Policy information about studies involving animals; ARRIVE guidelines recommended for reporting animal research

Laboratory animals

For zebrafish studies, fish larvae were obtained from established transgenic Tg(mpx:EGFP/flk:mCherry) stock lines housed at the NHGRI zebrafish core. Zebrafish were injected at 3 dpf and imaged between 3-4 dpf. Gonad differentiation to determine sex had not been completed by the time points used in these experiments. Fertilized White Leghorn chicken eggs were obtained from the University of Alberta Poultry Research Centre. For tail vein injection, five-to-seven-week-old female NOD-SCID Gamma (NSG) mice weighing 15-25 g were obtained from Johns Hopkins animal core facility. Animal rooms were maintained at 30-70% relative humidity and a temperature of 18-26ºC with a minimum of 10 room air changes per hour. Cages were changed once a week. Mice were fed a diet containing low fiber (5%), protein (20%) and fat (5-10%).

Wild animals

This study did not involve wild animals.

Field-collected samples

This study did not involve samples collected from the field.

Ethics oversight

Mice studies were conducted with all relevant ethical regulations outlined in protocols approved by the Johns Hopkins University Animal Care and Use Committee. Zebrafish studies were conducted under protocols approved by the National Cancer Institute and the National Institutes of Health Animal Care and Use Committee. Chick embryo studies were performed by following procedures approved by the University of Alberta Institutional Animal Care and Use Committee.

Note that full information on the approval of the study protocol must also be provided in the manuscript.

Flow Cytometry

Plots

Confirm that:

- [ ] The axis labels state the marker and fluorochrome used (e.g. CD4-FITC).
- [ ] The axis scales are clearly visible. Include numbers along axes only for bottom left plot of group (a 'group' is an analysis of identical markers).
- [ ] All plots are contour plots with outliers or pseudocolor plots.
- [ ] A numerical value for number of cells or percentage (with statistics) is provided.

Methodology

Sample preparation	All cell suspensions were filtered through a 40 μm cell strainer to remove cell aggregates and clumps; no staining was performed.
Instrument	Sony SH800 was used for cell sorting (with 100 μm sorting chip, 488 and 561 nm laser lines).
Software	Data were collected with Sony Cell Sorter Software (Version 2.1.5).
Cell population abundance	Cell population abundance: Arp3-pmCherryC1, beta-actin-mRFP-PAGFP or NHE1-GFP cells were collected. For sorting the beta-actin-mRFP-PAGFP cells, the red channel was used.
Gating strategy	Doublets and debris were rejected based on FSC and SSC characteristics.

- [ ] Tick this box to confirm that a figure exemplifying the gating strategy is provided in the Supplementary Information.
