# Supplementary material for: Extracellular fluid viscosity enhances cell migration and cancer dissemination
Source: Nature. 2022 Nov 2;611(7935):365–73. doi: 10.1038/s41586-022-05394-6 (PMC9646524; doi:10.1038/s41586-022-05394-6)
Supplement: Supplementary file 4 — A list of parameters and their values used in the two-phase model for confined cell migration. [file 41586_2022_5394_MOESM4_ESM.pdf]

Supplementary Information 4. Description of parameters and their values used in the model

Parameter	Description	Value	Source
R (J/mol/K)	Ideal gas constant	8.31	Constant
T (K)	Absolute temperature	310	Physiological condition
η (Pa s/ μm^2/mM)	Interfacial drag coefficient	$1\text{x}10^{-3}$	Based on[69]
$\eta^0_{\mathrm{st,LV}}$ (Pa s/ μm^2/mM)	Focal adhesion strength coefficient	800	Based on[62]
k_{σ_n} (Pa/mM)	Passive F-actin stress coefficient	100	Estimated
$J^{\mathrm{f}}_{\mathrm{actin}}$ (nm mM/s)	Coefficient of actin polymerization	2.5	Based on[62]
$\theta_{c,c}$ (μM)	Critical G-action concentration	0.2	Based on[70]
γ (1/s)	Constant rate of actin depolymerization	$6\text{x}10^{-4}$	Based on[71]
θ_* (mM)	Average actin concentration	300	Based on[70,72]
D_c (μm^2/s)	Diffusion coefficient of ion	100	Based on[21]
D_{θ_c} (μm^2/s)	Diffusion coefficient of G-actin	10	Estimated
$k^{\mathrm{f(b)}}_{\mathrm{sol}}$ (μm/s)	Passive ion channel permeability	50	Based on[62]
$J^{\mathrm{b}}_{c,\mathrm{active}}$ (μm mM/s)	Active ion flux at the back	−5300	Based on[62]
$\alpha^{\mathrm{f(b)}}$ (μm/Pa/s)	Water permeability	10^{-4}	Based on[62]
$p^{\mathrm{f(b)}}_0$ (Pa)	Hydraulic pressure at infinity	0	Free constant
$c^{\mathrm{f(b)}}_0$ (mM)	Extracellular ion concentration	340	Physiological condition

References

21 Stroka, K. M. *et al.* Water permeation drives tumor cell migration in confined microenvironments. *Cell* **157**, 611-623 (2014).

62 Li, Y., Yao, L., Mori, Y. & Sun, S. X. On the energy efficiency of cell migration in diverse physical environments. *Proc Natl Acad Sci U S A* **116**, 23894-23900 (2019).

69 Dembo, M. & Harlow, F. Cell motion, contractile networks, and the physics of interpenetrating reactive flow. *Biophys J* **50**, 109-121 (1986).

70 Pollard, T. D., Blanchoin, L. & Mullins, R. D. Molecular mechanisms controlling actin filament dynamics in nonmuscle cells. *Annu Rev Biophys Biomol Struct* **29**, 545-576 (2000).

71 Kuhn, J. R. & Pollard, T. D. Single molecule kinetic analysis of actin filament capping. Polyphosphoinositides do not dissociate capping proteins. *J Biol Chem* **282**, 28014-28024 (2007).

72 Satcher, R. L., Jr. & Dewey, C. F., Jr. Theoretical estimates of mechanical properties of the endothelial cell cytoskeleton. *Biophys J* **71**, 109-118 (1996).
